# The potential role of CGRP in synuclein-associated neurodegenerative disorders

**DOI:** 10.3389/fnins.2024.1479830

**Published:** 2024-11-06

**Authors:** Athanasia Alexoudi, Vincenzo Donadio, Elissaios Karageorgiou

**Affiliations:** ^1^Neurological Institute of Athens, Athens, Greece; ^2^IRCCS Institute of Neurological Sciences of Bologna, Bologna, Italy

**Keywords:** CGRP, synuclein-associated neurodegenerative disorders, neuroinflammation, GLP-1, apoptosis, neuromodulation

## Abstract

In this hypothesis article, the potential clinicopathological associations of Calcitonin Gene Related Peptide (CGRP) with the development of synuclein-associated neurodegenerative disorders (SAND) are discussed. The presence of *α*-syn and CGRP in the CNS and the ENS and the intricate role of CGRP and its related pathways in inflammation, apoptosis, metabolism, neuromodulation, and brain-gut communication are analyzed. Since this hypothesis is confirmed, modulating CGRP-potential related pathways may lead to novel disease-modifying therapies.

## 1 Introduction

### 1.1 Overview of SAND

SAND are defined by aggregates of abnormally misfolded *α*-synuclein (α-syn) in the peripheral and central nervous systems, which cumulatively refer to Parkinson’s disease (PD), Lewy body degeneration (LBD), and multiple system atrophy (MSA) pathologies (Lim et al., 2016). A special case of SAND is Pure Autonomic Failure (PAF) where *α*-syn aggregates are almost exclusively observed in the autonomic nerves of the peripheral nervous system. Differences in the macroscopic distribution and microscopic features of *α*-syn are associated with different clinical SAND phenotypes. Typical microscopic features in SAND are intracellular *α*-syn inclusions [Lewy bodies (LBs)] deposited in neurons [Lewy neurites (LNs)], present in LBD and PD, and also in brain glial cells and skin Remak non-myelinating Schwann cells in MSA (Lim et al., 2016; Donadio et al., 2023). Even more, dopaminergic neuronal loss in the substantia nigra pars compacta (SNpc) is observed across SAND, explaining common symptoms of parkinsonism. The aggregation and spread of *α*-syn are associated with disease severity and prognosis (Stewart et al., 2015; Lim et al., 2016), explaining the high morbidity and mortality (1.75–3.86 higher mortality risk in PD, 3.94 in LBD, and 10.51 in MSA) (Savica et al., 2017).

### 1.2 Role of neuroinflammation

For the past 30 years there is accumulating evidence that neuroinflammation is associated with the PD pathological processes, from which the following main narratives have emerged. First, early studies indicated the activation of microglial cells and infiltration of T lymphocytes in brain regions with pathological *α*-syn accumulation in patients with PD. Second, modified cellular immunity changes in monocytes and T lymphocytes have been described in peripheral blood, including increased phagocytic capacity, decreased effector CD8+ T cells and lower cytotoxicity natural killer (NK) cells. These inflammatory mechanisms have been supported by the increased expression of proinflammatory cytokines in blood, cerebrospinal fluid, and brain tissue. Third, examining the inflammasome’s integral role in inflammatory mechanisms, *α*-syn has been shown to contribute to inflammasome activation (Codolo et al., 2013). Fourth, genes mediating immune responses have been associated with presence of PD (including LRRK2, DJ-1, PINK1, GBA, SNCA, PARK2, MAPT, ER *β*, PDLIM2, STK39, DYRK1A), strengthening the neuroinflammation theory. The above, however, have not verified a specific pathophysiological sequence of events through which inflammation triggers PD (Kung et al., 2022). Considering that CGRP modulates lymphocyte activity, and that CGRP and its related pathways are known to contribute to inflammation in other conditions, we postulate that CGRP pathways are also critical in inflammatory-driven SAND pathology, although dedicated studies in SAND are lacking. As we expand on below, CGRP belongs to the calcitonin family of peptides and is widely expressed in neuronal tissue (Wimalawansa et al., 1990). CGRP receptor binding leads to activation of multiple signaling pathways with downstream effects, where their modulation could prove causal to neurodegeneration (Umoh et al., 2014). In the case of Alzheimer’s disease, it has been suggested that exogenous administration of CGRP inhibits tissue infiltration of macrophages and expression of various inflammatory mediators, which in turn attenuate inflammatory responses (Singh et al., 2017). Therefore, from a bird’s eye view, the potentially protective role of CGRP and its related pathways in SAND deserve further examination.

In this hypothesis article we will discuss the clinicopathological associations of Calcitonin Gene Related Peptide (CGRP) and synuclein-associated neurodegenerative disorders (SAND). CGRP is a key molecule in inflammatory processes, with the development of SAND, suggesting novel disease-modifying therapeutic pathways worth examining. This follows on recent research supporting neuroinflammatory processes as key players in the pathogenesis of SAND (Lim et al., 2016).

## 2 CGRP structure and physiologic pathways

### 2.1 CGRP structure

CGRP expression is abundant across tissues, including neuronal tissue (Russell et al., 2014). There are two major CGRP isoforms, *α* and *β*CGRP or CGRPI and II. They differ by only three amino acids in rats and by one in humans, and although they have similar structure and biological activities, they are synthesized from different genes at different sites on chromosome 11 (Wimalawansa et al., 1990). Functional comparisons indicate that αCGRP is severalfold more immunoreactive compared to its β isoform (Schulz et al., 1999; Schütz et al., 2004). αCGRP is formed via alternative splicing of the calcitonin/CGRP gene. It is widely expressed in neuronal tissue and is primarily localized on C and Aδ sensory fibers. βCGRP is produced from its own second human calcitonin gene (hCGRP-II, or CALC II). CGRP is a multifunctional peptide with high vasodilation potential (Russell et al., 2014). Historically, *α*CGRP has been presumed to exist in both the central and peripheral nervous systems (CNS and PNS), whereas βCGRP to exist predominantly in the enteric nervous system (ENS). It has become clear, however, that both isoforms are expressed in the CNS and ENS (Russell et al., 2014). This common theme of CGRP and pathologic α-syn being expressed in the CNS and the ENS was one of the triggers in examining their potential relationship.

The two isoforms bind to the CGRP receptor, which is composed of two subunits. The “calcitonin receptor-like receptor” (CLR), discovered in early 1990s, which is unresponsive to CGRP binding on its own (Chang et al., 1993; Russell et al., 2014). As such, another protein co-expressed with CLR is required to effect CGRP binding and is known as the “receptor activity modifying protein” (RAMP). Three RAMPs have been recognized (RAMP1, RAMP2, RAMP3). The resulting heterodimeric complex of CLR and RAMP in the cell membrane is stable. The receptor composed of CLR and RAMP1 has high affinity for CGRP. Instead, the CLR/RAMP2 (AM1 receptor) and CLR/RAMP3 (AM2 receptor) combinations respond to the adrenomedullin peptide, of which the AM2 receptor shows affinity for CGRP as well (Choksi et al., 2002). Even when CLR/RAMP are combined, the CGRP receptor is not functional without the mediation of a third protein; the receptor component protein (RCP). Although the absence of RCP has no effect on CGRP binding affinity, downstream signal transduction is attenuated (Evans et al., 2000).

### 2.2 Production and release of CGRP

Despite extensive research to date, the process of CGRP synthesis and release is not fully clarified. It is suggested that CGRP is produced in both the CNS and PNS, and its synthesis is promoted after nerve damage and inflammatory responses (Saito et al., 2022). In the CNS, it is suggested that CGRP is synthesized in the anterior horn of the spinal cord and the cell bodies of motor neurons (Evangelista, 2013). It is postulated that nerve growth factor (NGF) released from cells such as macrophages and keratinocytes, as well as other factors, such as brain-derived neurotrophic factor (BDNF), participate in the process (Salio et al., 2007). In the PNS, there is evidence that CGRP release is mediated through transient receptor potential vanilloid 1 (TRPV1, also known as the capsaicin receptor and the vanilloid receptor 1) and TRP ankyrin 1 (TRPA1) activity, which in turn are dependent on Protease Activated Receptor 2 (PAR2) activation during inflammatory processes (Saito et al., 2022). Once CGRP is produced, it is stored in vesicles located at the sensory nerve terminal (Matteoli et al., 1988). Besides peripheral neurons, different types of immune cells (activated B-lymphocytes, mononuclear cells, and macrophages) can produce CGRP, suggesting an additional possible mechanism of immune response down-regulation (Linscheid et al., 2004).

### 2.3 Receptor activation

**Figure 1:**
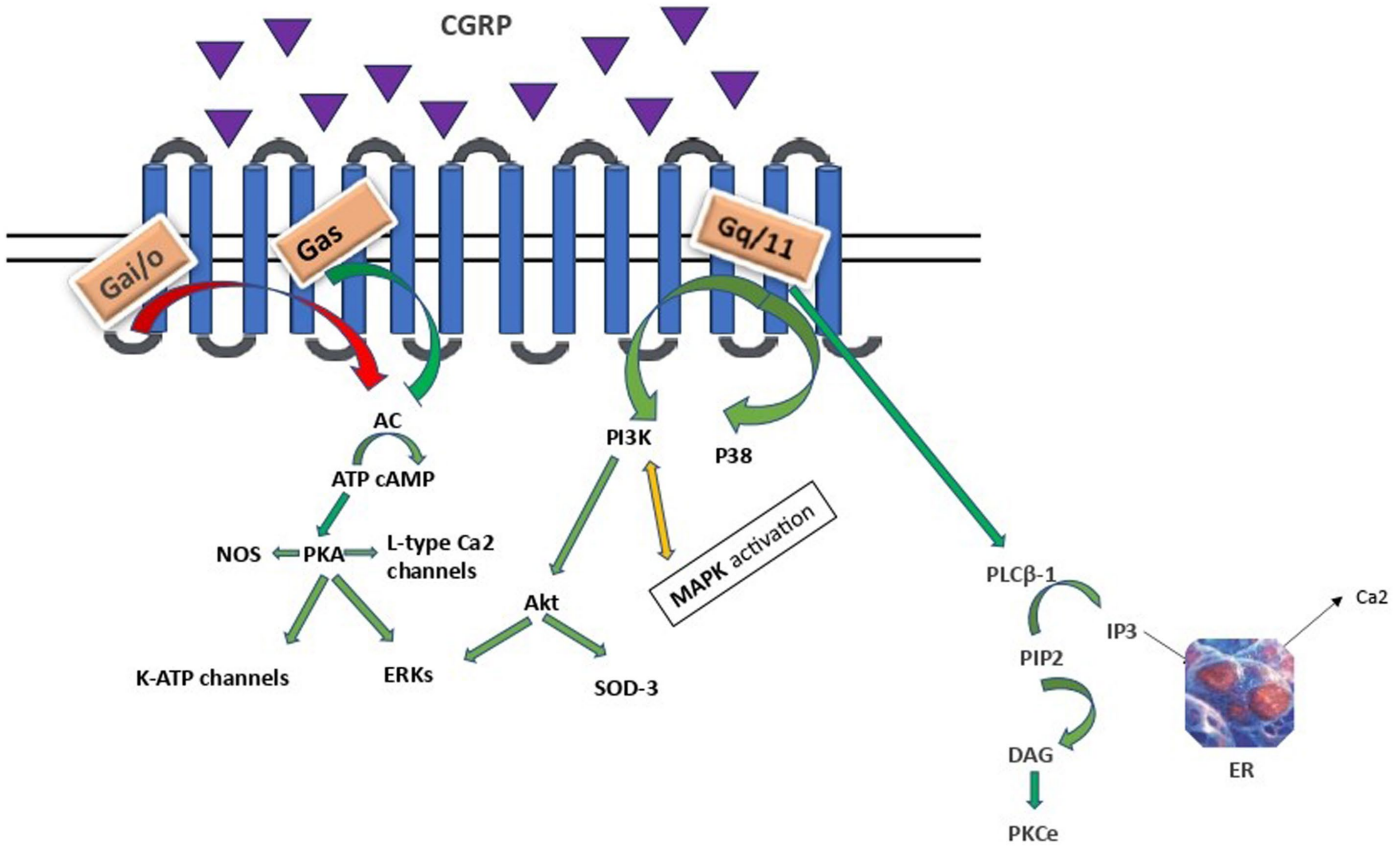
CGRP intracellular signalling. AC, adenylyl cyclase; cAMP, cyclic adenosine monophosphate; PKA, protein kinase A; ERKs, extracellular signal-related kinases; PLCB-1, phosphoinositide-phospholipase CB 1; PIP2, phosphatidylinositol 4,5-bisphosphate, IP3, inositol trisphosphate; DAG, diacylglycerol; PKCe, protein kinase C epsilon type, PI3K/Akt, Phosphoinositide 3- kinase/Protein kinase B; MAPK, mitogen - activated protein kinase; SOD3, superoxide dismutase 3; ER, endoplasmic reticulum. Arrows: green: facilitation, red: inhibition, orange: conflicting association.

Binding of CGRP to the CLR/RAMP1 receptor leads to receptor activation and coupling to Gαs, Gαi/o or Gαq/11 proteins resulting in downstream effects as depicted in Figure 1.

Gαs and Gαi families regulate adenylyl cyclase (AC) activity. The Gαs family stimulates, while Gαi/o exerts an inhibitory effect. Vasodilation, inflammation, and gene transcription are regulated through a direct G*α*s protein pathway (Saito et al., 2022; Rysiewicz et al., 2023). The CGRP receptor-induced crosstalk between PI3K/Akt and ERK, leading to decreased Akt, NFkB, and SOD-3, as well as increased ERK1/2 and p38 MAPK expressions, suggests that CGRP weakens anti-apoptotic and strengthens proliferative signaling pathways in an Akt/ERK-dependent manner (Umoh et al., 2014). Specifically, Akt regulates neuronal toxicity and mediates neuronal survival through the PI3K signaling pathway. ERK is a kinase and once phosphorylated can promote different processes in different cellular targets under particular conditions, such as neural differentiation, migration, apoptosis and neurogenesis. Apparently, PI3K/Akt and ERK pathways play opposite roles in the prevention of neuronal apoptosis. Besides, α-syn promotes inflammation via activating p38 and ERK in human microglial cells. Therefore, these proteins and their related pathways play a vital role in the pathogenesis of neurodegenerative diseases, including PD (Rai et al., 2019; Albert-Gascó et al., 2020). Finally, CGRP activation of the Gαq/11-dependent pathway generates inositol trisphosphate (IP3) and diacylglycerol (DAG). DAG activates and anchors PKC to the plasma membrane, whereas IP3 diffuses to the endoplasmic reticulum (ER), toward the release of calcium in the cytoplasm (Cottrell, 2019).

### 2.4 Internalization and desensitization

The aforementioned CGRP receptor signaling is modulated by CGRP internalization and CGRP receptor/b-arrestin complex desensitization and trafficking. Specifically, early studies on desensitization revealed that signaling was attenuated after a second exposure to CGRP, where the CLR component of the receptor is phosphorylated and internalized through the participation of arrestin, clathrin, and G protein–coupled receptor kinases (Walker et al., 2010).

Interestingly, there seem to be different acute and chronic effects of CGRP on inflammation. In acute peripheral inflammation, increased synthesis of CGRP is present, attenuating in chronic phase (Liu et al., 2012). We hypothesize that this difference is partially explained by the duration of CGRP receptor stimulation. Acute stimulation induces CLR/RAMP1 receptor recycling, correlated with resensitization, whereas chronic stimulation leads the CGRP receptors to degradation by lysosomes (Cottrell et al., 2007).

CGRP role as a biomarker in the field of pain is substantial. Cytokine activin C mainly expressed in small diameter dorsal root ganglion (DRG) neuron, significantly influences the release of calcitonin gene-related peptide (CGRP) in the nervous system, particularly following peripheral nerve injury. It appears to enhance CGRP release through its interaction with TRPV1, as evidenced by the loss of analgesic effects in TRPV1 knockout mice. The normalized data on CGRP support the idea that activin C plays a complex role in modulating chronic neuropathic pain, and its action is linked to inhibiting pro-inflammatory mediator-induced ERK phosphorylation. Moreover, activin C levels are reduced during early persistent inflammation, but its intrathecal application can effectively inhibit inflammation-induced hyperalgesia (Zhang et al., 1994; Liu et al., 2012; Huang et al., 2020; Chen et al., 2024).

## 3 Supposed CGRP mechanisms involved in sand

**Figure 2:**
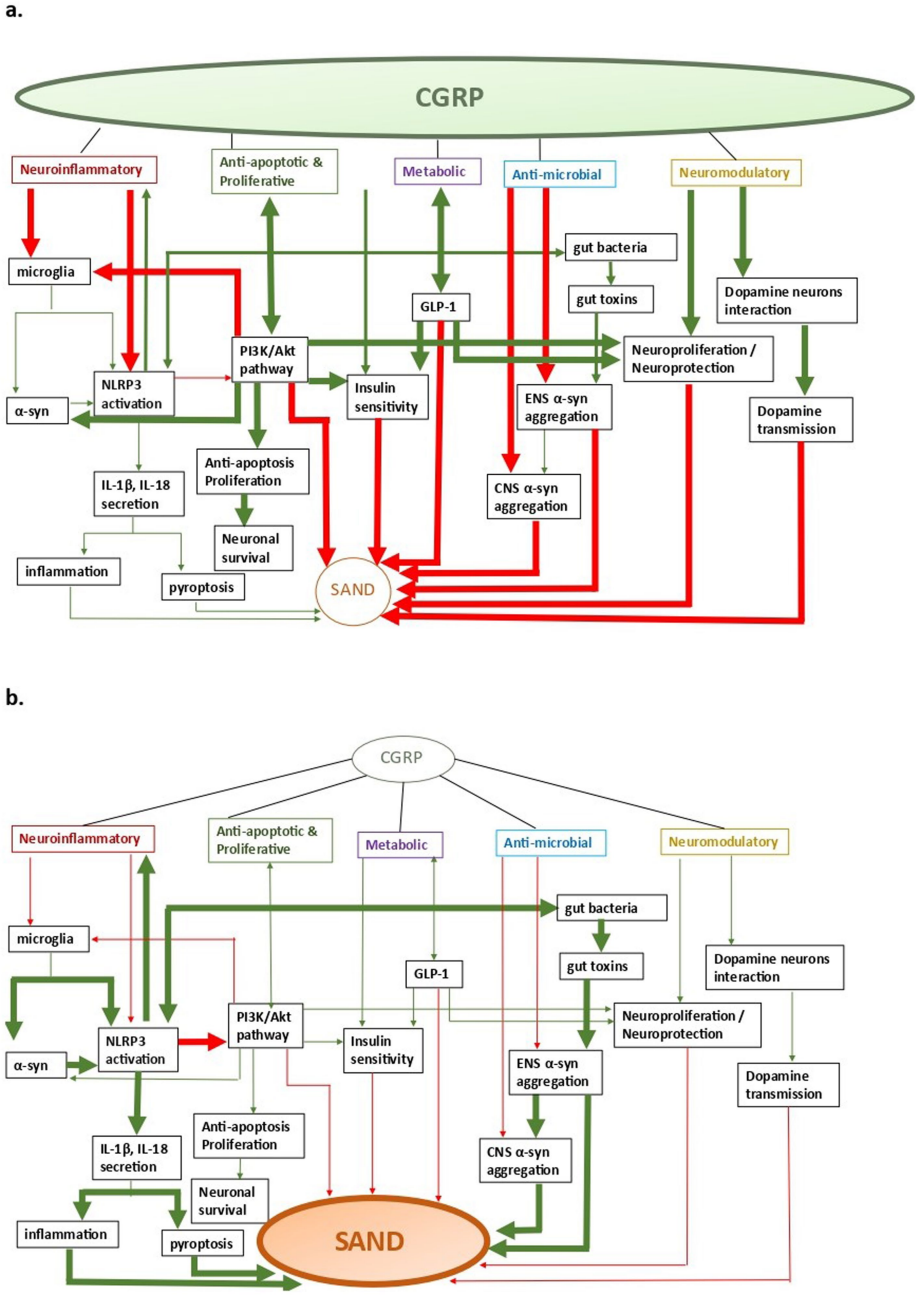
CGRP-related pathways in SAND; a. CGRP-pathway activation, b. SAND evolution (a) neuroinflammatory, (b) anti-apoptotic and proliferative, (c) metabolic, (d) anti- microbial, and (e) neuromodulatory. Arrows: green: facilitation, red: inhibition.

There are five main CGRP pathophysiological mechanisms that can have an effect on SAND pathology (Figure 2): (a) neuroinflammatory, (b) anti-apoptotic and proliferative, (c) metabolic, (d) neuromodulatory, and (e) anti-microbial. Of these mechanisms, most data explain CGRP-related neuroinflammatory processes in SAND, which also overlap with the other mechanisms. Although there is evidence of potentially beneficial treatments across specific CGRP pathways, targeting CGRP pathways upstream may allow for more impactful multi-pathway effects as we discuss below.

### 3.1 CGRP, Neuroinflammation, and SAND

A key mechanism through which CGRP mediates its effect is by modulating neuroinflammatory pathways. Neuroinflammation has been implicated in the triggering and evolution of pathological changes in many neurodegenerative diseases, including PD (Lim et al., 2016). Key inflammatory processes involve the excessive activation of microglia, especially through NOD-, LRR-, and pyrin domain-containing protein 3 (NLRP3) inflammasome pathways, which have been observed in tissues collected from patients with SAND (Haque et al., 2020). It follows that downstream anti-inflammatory interventions have been considered in delaying PD progression, albeit without any definite benefits from clinical trials to-date (Hung and Schwarzschild, 2020). Within this framework, studies support CGRP having anti-inflammatory properties through CD8 proliferation, and NK killing activity inhibition, production of mononuclear phagocyte promotion, macrophage regulation, especially microglia, and NLRP3 inflammasome inhibition (Zhu et al., 2023).

#### 3.1.1 Microglia in SAND

**Figure 3:**
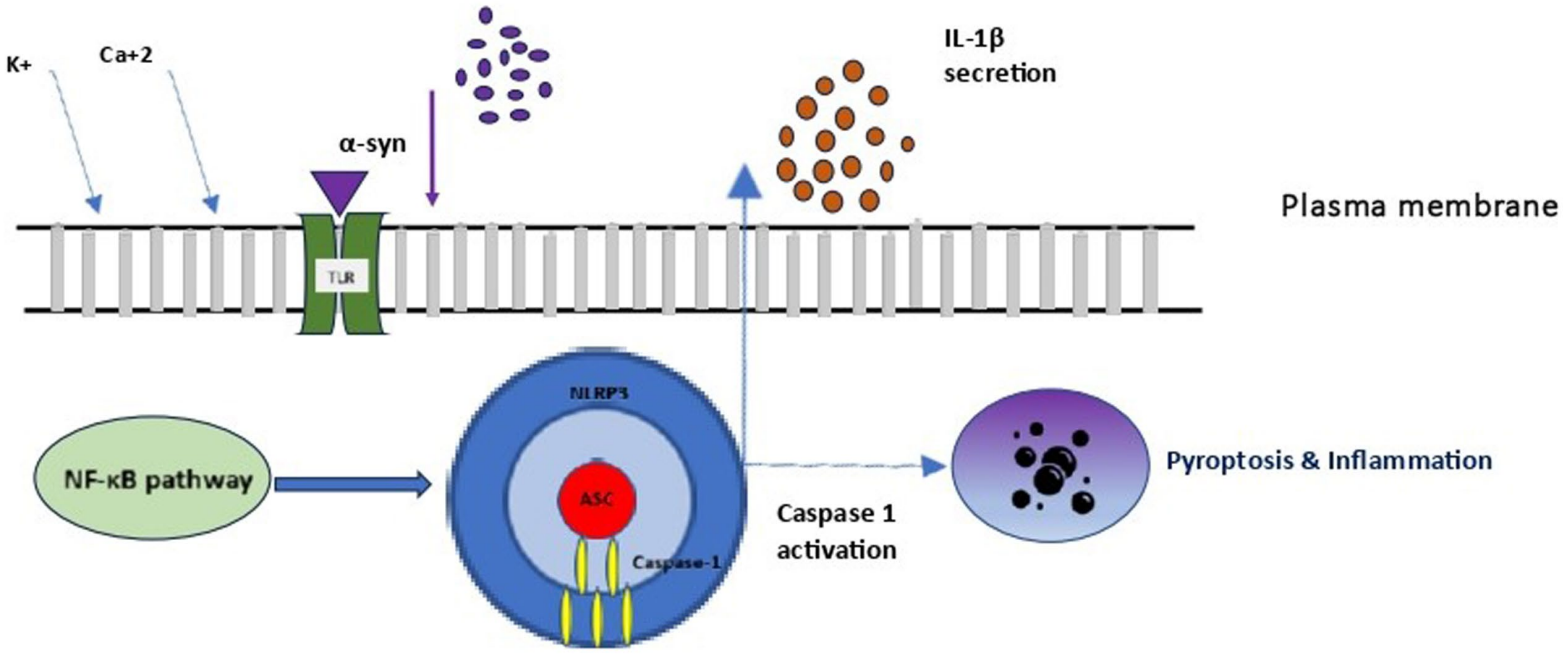
Microglial NLRP3 inflammasome activation. NLRP3, NOD-LRR- and PYD-containing protein 3; PC1: procaspase 1; ASC, apoptosis associated speck-like protein containing a CARD.

Microglia, the resident immune cells of the brain, fulfil a variety of different tasks within the central nervous system (CNS) and display several different characteristics across resting (non-phagocytic) and activated (phagocytic) states (Wake et al., 2009). Resting microglia contact neuronal synapses, undergo transformation similar to that of macrophages, and respond to stimuli such as neuronal death (Wake et al., 2009). The activation of microglia through distinct signaling pathways is responsible for both their beneficial effect on clearance and blocking of detrimental inflammation, as well as for cascades contributing to neurodegeneration. This differential modulation of microglia explains existing controversies on their role in neurodegeneration (Lim et al., 2016). Research over the last decade revealed their contribution as both an active and re-active player in neurodegeneration, highlighting the significance of developing selective microglia-mediated treatment interventions that either inhibit or enhance neurodegenerative pathways, rather than pursuing non-specific inhibition of microglia. Microglia are activated as a response to extracellular stimuli, including endotoxins, cytokines, chemokines, mis-folded proteins, and ATP. With regards to SAND, it is possible that microglia activation follows systemic infection (e.g., Western equine encephalitis virus, Japanese-encephalitis, bacterial infections) or exposure to factors which promote oxidative stress (Wake et al., 2009). Beyond the aforementioned stimuli, microglia are triggered from *α*-syn into activating inflammatory pathways. Specifically, α-syn exists either as monomeric or fibrillar form in the brain. Under certain conditions, α-syn secretion leads to the increase of misfolded and/or aggregated proteins, which have been typically associated with direct neurotoxicity via impaired vesicle recycling, endoplasmic reticulum transport, mitochondrial energy production, and protein degradation (Haque et al., 2020). In addition to direct α-syn neurotoxicity, an indirect α-syn-induced neurodegeneration pathway involves microglial activation towards inflammation. In this pathway, neuron-secreted α-syn binds on microglial Toll-like receptor 2 (TLR2), facilitating their activation with secretion of cytokines and chemokines, leading to inflammation and neurotoxicity (Figure 3). Additionally, TLR4 is also involved in these pathways, where it has been suggested it mediates both microglial and astrocytic α-syn-dependent activation toward α-syn clearance (Fellner et al., 2013). Subsequent to α-synuclein uptake, multiple inflammatory signaling pathways are activated in microglia, with more prominent the critical dopamine receptor- mediated NLRP3 inflammasome pathway, which leads to neuroinflammation and subsequent neurodegeneration. Across cells, microglia and peripheral macrophages are the main cell types responsible for inflammasome activation, further explaining their critical role in inflammation-mediated disorders (Martinon et al., 2002). Considering the above microglial effects on SAND, study results suggest that exogenous administration of CGRP inhibits infiltration of macrophages and expression of various inflammatory mediators, raising the possibility of CGRP receptor stimulation becoming a potential target for SAND therapy (Singh et al., 2017).

#### 3.1.2 The role of the inflammasome in SAND

Inflammasomes are a group of cytosolic multiprotein complexes of the innate immune system that activate inflammatory caspases, allowing for the release of cytokines and alarmins into circulation and inducing pyroptosis, a type of inflammatory programmed cell death. Each inflammasome consists of three components (Figure 3): (1) a nucleotide-binding domain and leucine-rich repeat receptor (NLR) [or an absent in melanoma 2-like receptor (ALR) in the special case of melanoma], (2) an adaptor component named Apoptosis-Associated Speck-like Protein Containing a Caspase Activation and Recruitment Domain (ASC), and (3) an effector component named pro-caspase 1. The inflammasome complex achieves cytosolic sensing of pathogen (or danger)-associated molecular patterns via the NLR (or ALR). At least five different types of NLRs have been recognized as inflammasome components, and naming of an inflammasome has conventionally been defined by their NLR component. NLR protein complexes (NLRP) have different subtypes depending on the differential combination of their components. Of all NLRPs, NLRP3 is the best studied inflammasome involved in human diseases. The ASC is usually recruited by activated NLR (and ALR) toward engaging caspase-1 activation. Finally, pro-caspase 1 activation to caspase 1 leads to the secretion of proinflammatory cytokines (Martinon et al., 2002). Activation of the inflammasome is responsible for inflammatory responses by promoting proteolytic cleavage, maturation, and secretion of pro-inflammatory cytokines interleukin 1β (IL-1β) and IL-18, and eventually pyroptosis (Figure 3) (Martinon et al., 2002).

When examining the inflammasome role in SAND, monomeric and fibrillar *α*-syn released by degenerating neurons into the extracellular space are first recognized by microglial TLR, and thus activate the NF-κB pathway and the production of IL-1β precursor protein. However, only fibrillar α-syn induces the NLRP3 response, which acts as an endogenous trigger that activates microglial cells and induces a strong inflammatory response and pyroptosis in SAND (Codolo et al., 2013). In a vicious cycle, the extracellular secretion of mature IL-1*β* leads to inflammatory damage and death to dopamine neurons in the substantia nigra, as demonstrated in a murine model (Ferrari et al., 2006). Although the exact pathophysiology of this process is unclear, the secretion of mature IL-1β by the activated inflammasome leads to pyroptosis, during which, IL-1β is also secreted, further accentuating inflammation. To the above microglial-mediated process, both astrocytes and peripheral immune cells that enter the central nervous system participate in and amplify the core microglial-mediated inflammatory response. MCC950 is a well-known NLRP3 inhibition factor, which suppressing NLRP3 activity allows inflammation reduction and neurons’ protection in the substantial nigra in experimental models (Cheng et al., 2020).

Studies from the field of Diabetes Mellitus (DM) directly link NLRP3 and CGRP-related pathways, where NLRP3 activation (e.g., via reactive oxygen species) and consequent IL-1β production have been shown to affect receptor resistance and apoptosis in DM models (Guo et al., 2015). It has been demonstrated that IL-1β activation leads to disrupted PI3K-Akt signaling, as well as increased TNF-*α*, in insulin-targeted cells by decreasing insulin sensitivity and inducing JNK-dependent serine phosphorylation of insulin receptor substrate-1 (IRS-1) (Wen et al., 2011). IL-1β also leads to pancreatic β-cell loss in DM. The inverse effects have been observed in animal models exposed to a high-fat diet where deficiency of NLRP3, ASC, and/or caspase-1 lead to reduced inflammatory cytokine levels, increased insulin-PI3K-Akt signaling, and improved glucose tolerance and insulin sensitivity (Vandanmagsar et al., 2011; Wen et al., 2011). CGRP is a B-cell growth factor and probably an inhibitory factor for insulin secretion (Manaka et al., 1998). Additionally, as we discussed above, CGRP is known to lead to similar downstream effects in PI3K-Akt signaling, but there are no known studies in SAND on dopaminergic neurons or α-syn pathology. By extension, the fact that patients with DM are 40% more likely to develop PD is further suggestive of their common pathophysiological pathways, including inflammasome activation.

Overall, a crosstalk between CGRP and NLRP3 seems to be at play. The NLRP3 inflammasome triggers the CGRP increase and therefore blockade of NLRP3 or IL-1β reduces the upregulation of CGRP and attenuates expression of the neuronal activation marker p-ERK in the CNS (He et al., 2019). Conversely, CGRP directly inhibits NLRP3 activation, as typically seen in bacterial infections. Cytosolic CGRP binds directly to NLRP3 and inhibits any downstream inflammatory response (Zhu et al., 2023).

A more recently identified mechanism through which NLRP3 participates in inflammation is through the gut-brain axis, where NLRP3 is modulated by gut bacteria. It has been proposed that altered composition of enteric microbiota may lead to dysbiosis and promote inflammation via gut-brain communication. The involvement of the enteric plexus in PD justifies further studies to assess if an altered microbiome truly contributes to the degenerative process.

In a more traditional approach, the close relationship between inflammasome activation and SAND is corroborated by a series of studies that examined biosamples from patients with PD. Specifically, NLRP3 has comparatively higher activation in serum samples of patients with PD compared to controls. This activation is modulated by genetic variations of NLRP3 single-nucleotide polymorphisms (SNPs), impacting the progression of PD (von Herrmann et al., 2018). In keeping with the above, patients with PD also have higher levels of IL-1β and IL-18 in serum and cerebrospinal fluid compared to controls (Zhang et al., 2016). Even more, and of clinical significance, higher levels of IL-1β and total plasma *α*-syn have been associated with worse motor function in PD (Fan et al., 2020). Beyond PD, the density of microglia expressing NLRP3 inflammasome-related proteins have been significantly upregulated in the putamen of patients with MSA, and correlate with severity of neurodegeneration (Li et al., 2018).

Considering the above, the NLRP3 inflammasome and its related pathways are attractive therapeutic targets in SAND by mitigating neuroinflammatory effects and delaying progression of synuclein-associated pathology. Indeed, there are already a couple of key observations of inflammasome regulation with potential therapeutic implications. First, NLRP3 activity is attenuated by activation of the dopamine D1 receptor cyclic adenosine monophosphate (cAMP) signaling pathway in primary microglia and astrocytes (Yan et al., 2015). The second mechanism is via caspase-1 deficiency, an important component of the NLRP3 inflammasome, which inhibits α-syn-induced microglial activation, resulting in neuroprotection of mesencephalic dopaminergic neurons, as observed in a murine model (Zhu et al., 2023).

Taking all these together, one may hypothesize that a neurotropic pathogen infection enhances neuronal expressions of CGRP. CGRP is overexpressed in order to impede the aggregation of α-synuclein. Once PD manifests clinically, the loss of CGRP immunoreactive neurons might be an additional pathological finding further to a reaction to the accumulation of pathological synuclein (Figure 2). This hypothesis might offer a possible causal link between PD and CGRP, which however needs to be further investigated. Studies have revealed elevated neocortical pro- and anti-inflammatory cytokines in AD, but not in late-stage LBD. Furthermore, significant CGRP neuronal loss has been observed in the parabrachial nucleus (PBN) and Kolliker-Fuse (KF) nucleus in progressed MSA. These findings are in keeping with our model, where the inflammatory process is more pronounced at the beginning stages of the disease and then is attenuated over time.

### 3.2 CGRP anti-apoptotic and proliferative effects

Beyond anti-inflammatory mechanisms that are better studied in CGRP-related pathways, CGRP-mediated activation of the Akt/ERK signaling kinase cascade leads to both neurotoxic and neuroprotective downstream effects. The neuroprotective role of CGRP in multiple neuronal populations against neurotoxicity is mediated through pathways whose disruption affects neuronal cell proliferation, differentiation, and survival, thus causing neurodegeneration (Abushik et al., 2017). A key mechanism in this process is apoptosis, which is also a main mechanism of neuronal death in PD. Thus, treatments against apoptosis have been considered in SAND (Schulz et al., 1999). One such therapeutic target involves stimulation of a proliferation signaling cascade to promotes neuronal survival and increase neurite outgrowth and regeneration. There is evidence that CGRP weakens anti-apoptotic and strengthens proliferative pathways in an Akt/ERK- dependent manner (Umoh et al., 2014).

The PI3K-AKT pathway promotes survival and development of dopamine neurons by suppressing apoptosis via inhibition of B cell lymphoma 2 survival protein (Bcl-2)-antagonist of cell death. During the pathogenesis of PD, activation of the multifunctional serine/threonine protein kinase GSK-3 leads to apoptosis of dopaminergic neurons. Noteworthy, the same pathway inhibits GSK-3. Moreover, AKT and phosphorylated AKT are significantly decreased in the SNpc of patients with PD.

Finally, within the premise of its anti-apoptotic neuroprotective effects, CGRP reduced mitochondrial toxicity of the apoptosis-inducing toxin N-methyl-4-phenylpyridinium (MPP+) and protected a midbrain subpopulation of *α*-synuclein overexpressing PC12 dopamine cells *in vitro* (Chung et al., 2005). PC12 is a cell line that synthesizes, releases and stores catecholamines (Greene and Rein, 1977; Greene and Tischler, 1976). They are sensitive to mitochondrial toxins, such as 1-methyl-4-phenylpyridinium (MPP+), and rotenone (Nakamura et al., 2000; Hirata and Nagatsu, 2005). Therefore, it could be suggested even a potential neuroprotective effect of CGRP in adult dopamine neurons *in vivo*.

### 3.3 CGRP metabolic mechanisms in SAND

Another avenue through which CGRP-related pathways may mediate protective effects in SAND is metabolism. Beyond the inflammatory common pathways between DM and SAND, the two also share common metabolic pathways that engage additional processes.

One such mechanism involves Glucagon-like peptide effects, where it has been demonstrated in animal models that long-acting CGRP analogues have potential therapeutic benefits via positive metabolic effects of Glucagon-like peptide-1 (GLP-1) secretion (Nilsson et al., 2016). At the same time, GLP-1 receptor activation has been shown to increase gene expression of energy balance regulating peptides, IL-6, and CGRP within the parabrachial nucleus (Richard et al., 2014). Thus, there is a positive feedback pathway between GLP-1 and CGRP, that is regulated by a yet unknown mechanism. Metabolic benefits are further supported through a study in humans indicating that use of Dipeptidyl peptidase 4 (DPP4) inhibitors and/or GLP-1 mimetics are associated with a lower rate of PD compared to other oral antidiabetic drugs (Brauer et al., 2020). Whether this benefit hinges on people having DM rather than people without DM, and, even more, people who have already developed PD or other SAND, remains to be seen.

Another metabolic pathway involves fatty acids, which are involved in both inflammatory and energy metabolism processes. Specifically, saturated fatty acids induce NLRP3 inflammasome activation (Vandanmagsar et al., 2011). Additionally, *α*-synuclein highly co-localizes with fatty acid-binding protein (FABP) and dopamine long-type Dopamine-2 receptors, leading to impaired dopamine production via mitochondrial dysfunction. Instead, inhibition of FABP prevents FABP-induced neurotoxicity (Kawahata and Fukunaga, 2022).

The above raise the possibility that CGRP effects on downstream metabolic pathways may exert benefits in SAND. Nevertheless, the above conjecture is incomplete and should be interpreted with caution, since in a high-fat diet mouse model of DM, anti-*α*CGRP drugs reduced adiposity, albeit without changes on glucose homeostasis (Halloran et al., 2020).

### 3.4 CGRP-mediated neuromodulation

One of the most intriguing, and less understood, mechanisms CGRP-related pathways may be involved in SAND is neuromodulation. The neuromodulating role of CGRP is supported by its interaction with the dopaminergic system within the CNS. The high CGRP receptor densities that have been found in many brain regions, including basal ganglia, cortex, hippocampus, thalamus, hypothalamus, pituitary, and amygdala, facilitate the pervasive effects on dopamine transmission in these areas and modulate the activity of specific dopaminergic neurons that innervate selective dopamine terminal field regions (Deutch and Roth, 1987).

The overall concentration of CGRP in brain is fluctuating. It has been noted that CGRP expression is markedly increased by stress (e.g., injury, ischemia, hyperthermia, seizures, toxins) (Bulloch et al., 1996, 1998; Harada et al., 2009; Hashikawa-Hobara et al., 2015; Liu et al., 2010; Sharma et al., 2000; Zhang et al., 2010). Additionally, CGRP promotes neuroprotective/ neurotrophic processes via insulin-like growth factor-1 (IGF-1), basic fibroblast growth factor (bFGF), nerve growth factor (NGF), and strengthens antiapoptotic signaling via the Akt/ERK- pathway, cyclic AMP response element-binding transcription factor (CREB), and B cell lymphoma 2 survival protein (Bcl-2) (Umoh et al., 2014).

It seems these processes are activated by CGRP, toward ensuring cell survival and preventing apoptosis and neuronal death.

### 3.5 CGRP-microbiome interactions across the brain-gut axis

An association between CGRP and SAND is also suggested by the observed co-expression of *α*-syn and CGRP in the CNS and the ENS, especially given the hypothesis over the past two decades that ENS involvement may also be critical in the initiation and spread of SAND. This brain-gut axis hypothesis has opened interesting perspectives in the pathogenesis of neurodegenerative diseases, especially SAND. It has been suggested that gut microbial toxins may induce the production of *α*-syn aggregates in the ENS, which can be subsequently propagated to and proliferated in the CNS in a prion-like manner through the vagus nerve (Santos et al., 2019). Considering that germ-free mice have higher levels of CGRP, it is reasonable to postulate that, if α-syn aggregation in the ENS is secondary to microbial toxins, this effect is mediated by inhibiting CGRP and its pathways (Pujo et al., 2023). The inverse is also reasonable given the above, i.e., that CGRP-pathway activation protects from α-syn aggregation, since CGRP also has antimicrobial effects, but this and, more importantly, protection of dopamine network function, remain to be seen.

Additional support to a gut-brain axis mechanism is provided by studies where intestinal infection with potential pathogens (e.g., *H. pylori*, small intestinal bacterial overgrowth) is not only associated with worse motor fluctuations but may also contribute to the pathogenesis of the disease (Fasano et al., 2015). *H. pylori* infection contributes to neurodegeneration with different mechanisms. *H. pylori* disrupts the equilibrium of gastrointestinal microbiota with an excessive growth of small intestinal bacterial overgrowth. Besides the dysbiosis, the autotoxin produced by *H. pylori* induces pro-inflammatory cytokines release, thereby facilitating the occurrence of CNS inflammation through microbiome-gut-brain axis, leading to neuronal damage (Wei et al., 2024). Adding one more piece to the puzzle, CGRP expression is increased in the gastric mucosa and in the dorsal horn of the spinal cord of mice inoculated with *H. pylori*, further supporting our hypothesis that a pathogenetic factor (e.g., *H. pylori* infection), upregulates CGRP pathways both in the intestine and in the CNS, and thus makes CGRP a neuroprotective candidate in the neurodegenerative process (Li et al., 2009).

Neuroimmune interactions are vital for intestinal tissue homeostasis and host defense, yet the specifics remain unclear. Using a chemogenetic approach, eight distinct neuronal subsets were activated, revealing differential immune responses. Notably, nociceptor neurons expressing TRPV1 induced extensive immunomodulation by altering innate lymphocytes, macrophages, and RORγ+ regulatory T (Treg) cells. Specifically, TRPV1+ neurons in the dorsal root ganglia decreased Treg cell numbers through the neuropeptide CGRP. This finding suggests a significant link between pain signaling and the regulation of gut Treg cell function, highlighting CGRP’s critical role in neuroimmune crosstalk (Zhu et al., 2024).

Finally, another possible bidirectional brain-gut route of pathological α-syn spread between the CNS and the ENS is via the general circulation. Following on the selective vulnerability hypothesis, where certain areas of the nervous system are key hubs from where neurodegenerative diseases start and spread from (e.g., via prion-like mechanisms), the above can explain the differential pathology burden between PD, LBD, and MSA. In this model, the differential distribution of CGRP in both the brain and gut raises the possibility that it may play a role in this brain-gut relationship in SAND. Although interesting, especially for certain SAND phenotypes, adequate data to verify the above hypotheses are lacking.

## 4 CGRP-associated pathways as novel therapeutic targets in SAND pathogenesis

### 4.1 Potential therapeutic strategies

The above mechanisms justify targeting CGRP-related pathways in treating SAND. Overall, their disruption promote a number of pathophysiological conditions that are linked to SAND (Figure 2) (Russell et al., 2014). CGRP has a neuromodulating role facilitating the pervasive effects on dopamine transmission in many brain areas and modulating the activity of specific dopaminergic neurons which innervate selective DA terminal field regions (Deutch and Roth, 1987). It has been shown that CGRP and its receptor modulate the PI3K/Akt signaling pathway (Umoh et al., 2014). Additionally, CGRP is related to the regulation of macrophage polarization and the reduction of the NLRP3 and IL-1β in animal models, indicating that the molecule may alleviate inflammatory reactions (Zhu et al., 2023). The use of GLP-1 mimetics is associated with a lower rate of PD compared to the use of other oral antidiabetic drugs (Brauer et al., 2020). Long-acting CGRP analogues cause a specific concentration dependent CGRP-induced increased GLP-1 secretion, reduction in fasting insulin levels, a tendency to reduce fasting blood glucose and glycosylated hemoglobin, a decrease in food intake, and a significant decline in body weight in diet-induced obese rats. Thus, CGRP may have a therapeutic potential for the treatment of type 2 diabetes through positive metabolic effects and effect on GLP-1 secretion (Nilsson et al., 2016).

### 4.2 Existing and emerging therapies

The multiple potential neuroprotective effects activating CGRP-related pathways are likely to benefit neurological disorders beyond SAND. Nonetheless, GLP-1 analogs (i.e., exenatide and liraglutide) have shown potent neuroprotective activity in clinical and preclinical studies of PD. The therapeutic potential of the GLP1 analogues is mainly mediated through the involvement of PI3K/ERK/MAPK and PI3K/AKT-dependent pathways in terms of evading apoptosis toward cell survival (Athauda and Foltynie, 2016).

At the same time, these therapeutics may also increase the risk for other disorders by attenuated modulation of CGRP pathways. After all, it may be that optimized dynamic fluctuations of such complex pathways allow for homeostasis and cell survival, and, in the end, we may require to consider such chronotherapy dimensions when implementing novel treatments. Additionally, treatments for other conditions may be contributors to SAND evolution. A prime example is migraine and its novel therapies that suppress CGRP and improve migraine symptoms. Currently, a direct connection between PD and migraine has not yet been established and it is also unknown if anti-CGRP therapies increase the risk for developing SAND.

In the “AGES-Reykjavik Study” midlife migraine has been correlated to late-life parkinsonism. The results imply that there is a link between migraine with multiple indicators of parkinsonism (Scher et al., 2014).

In summary, CGRP-related pathways could prove to be novel targets in PD and other SAND therapeutics, as they contribute not only with their anti-inflammatory and anti-apoptotic properties, but also their positive metabolic effects on GLP-1 secretion, neuromodulation, and brain-gut communication. Clinical trials will have to be considered across both (a) upstream targets that mediate effects across multiple pathways, but also risk more widespread side effects, as well as (b) downstream specific CGRP-related pathway targets that engage fewer mechanisms and, thus, lead to fewer adverse events, but are more likely to have smaller effects.

## 5 Conclusion and future directions

### 5.1 Summary of findings

The presence of *α*-syn and CGRP in the CNS and the ENS and the intricate role of CGRP in inflammation, apoptosis, metabolism, neuromodulation, and brain-gut communication triggered our investigation of the possible association of CGRP-related pathways to alpha-synuclein aggregation and SAND. Prominent among its effects is CGRP regulation of multiple- complex signaling pathways, including PI3K/ERK/MAPK and PI3K/AKT, which in turn decrease inflammation and increase anti-apoptotic effects. There are already drugs, including anti-migraine targeting the CGRP pathway (i.e., anti-CGRP Monoclonal Antibodies and Gepants) in the market that could be candidates that effect upstream or downstream CGRP-related pathway effects on the evolution of SAND (Khan et al., 2019; Dodick et al., 2023; Haanes and Edvinsson, 2023; Reuter, 2023). However, despite the above suggesting that dynamic modulation of CGRP pathways can contribute to SAND, concrete causal associations are still and their circadian lacking to support our model as necessary or sufficient, nor does one specific target stand out compared to others as a better candidate for treatments.

### 5.2 Ongoing investigations and implications for future research

Given that many of the above hypotheses are extrapolations from other neurodegenerative disorders and pathological processes, we are currently pursuing a study supported by the Alzheimer’s Association, and planned to be completed in late 2025, on CGRP-related pathways in the development and progression of Clinicopathologically-established Parkinson’s Disease (CGRP in CPPD), in order to better establish whether CGRP and its downstream effects are indeed associated with PD pathology across dynamic (sleep–wake) cycles and across disease stages. Through this study we examine through a novel clinicopathological protocol in people with PD and non-impaired controls the unexplored cross-sectional and dynamic associations of blood and skin biopsy CGRP-related pathway biomarkers to multidimensional real-world data. Specifically, in the CGRP in CPPD study we will: (a) explore whether CGRP and inflammatory biomarkers (i.e., NLPR3 inflammasome, IL-1ß and IL-18) are associated to quantified phosphorylated a-synuclein in plasma and skin biopsy samples and their prognostic accuracy in CPPD clinical and pathological progression, (b) examine CGRP-related pathway associations dynamic fluctuations with regards to motor, cognitive, sleep features, cross-sectionally and longitudinally, and (c) investigate the association between medications known to modulate CGRP-related pathways and clinical and pathological CPPD progression. The results of the study will inform the extent of CGRP-related pathway contribution to PD across disease stages, and guide on possible specific therapeutic targets.

## Data Availability

The original contributions presented in the study are included in the article/supplementary material, further inquiries can be directed to the corresponding author.
